# Transmission of Avian Influenza A Viruses among Species in an Artificial Barnyard

**DOI:** 10.1371/journal.pone.0017643

**Published:** 2011-03-31

**Authors:** Jenna E. Achenbach, Richard A. Bowen

**Affiliations:** 1 Department of Microbiology, Immunology and Pathology, Colorado State University, Fort Collins, Colorado, United States of America; 2 Department of Biomedical Sciences, Colorado State University, Fort Collins, Colorado, United States of America; University of Georgia, United States of America

## Abstract

Waterfowl and shorebirds harbor and shed all hemagglutinin and neuraminidase subtypes of influenza A viruses and interact in nature with a broad range of other avian and mammalian species to which they might transmit such viruses. Estimating the efficiency and importance of such cross-species transmission using epidemiological approaches is difficult. We therefore addressed this question by studying transmission of low pathogenic H5 and H7 viruses from infected ducks to other common animals in a quasi-natural laboratory environment designed to mimic a common barnyard. Mallards (*Anas platyrhynchos*) recently infected with H5N2 or H7N3 viruses were introduced into a room housing other mallards plus chickens, blackbirds, rats and pigeons, and transmission was assessed by monitoring virus shedding (ducks) or seroconversion (other species) over the following 4 weeks. Additional animals of each species were directly inoculated with virus to characterize the effect of a known exposure. In both barnyard experiments, virus accumulated to high titers in the shared water pool. The H5N2 virus was transmitted from infected ducks to other ducks and chickens in the room either directly or through environmental contamination, but not to rats or blackbirds. Ducks infected with the H7N2 virus transmitted directly or indirectly to all other species present. Chickens and blackbirds directly inoculated with these viruses shed significant amounts of virus and seroconverted; rats and pigeons developed antiviral antibodies, but, except for one pigeon, failed to shed virus.

## Introduction

Gaining a more detailed understanding of the transmission potential of different avian influenza viruses (AIVs) among co-habitating species will enhance our ability to develop accurate models for disease spread, develop control strategies and, in some cases, assess risk of transmission to humans. Influenza A viruses are a common concern among many animal species including, birds, horses, pigs, sea mammals and humans, as the effects can range from asymptomatic to severe respiratory distress leading to death. While AIVs are maintained in wild water birds, they occasionally spread to other animals and humans and can lead to public health concerns, as currently is the case for highly pathogenic H5N1 viruses. All 16 hemagglutinin (HA) and 9 neuraminidase (NA) subtypes of influenza A virus are found in wild waterfowl, gulls, and shorebirds [1], [2], [3], but a much more restricted subset of these viruses is found in other birds and mammals.

Most strains of AIV are designated low pathogenic (LP) and cause minimal illness in chickens, as well as in wild waterfowl and shorebirds, but infection results in high levels of virus shedding, efficient spread among susceptible hosts, and perpetuation of the agent. Other AIV strains are classified as highly pathogenic (HP) and are restricted to members of the H5 and H7 subtype. HPAIV classification comes from the ability to cause severe morbidity and mortality in domestic fowl and more recently has caused mortality in wild waterfowl, mammals, and humans [4], [5]. In several outbreaks of HPAIV, circulation of a H5 or H7 LPAIV was detected shortly before the HPAIV outbreak of same subtype, and was determined to have evolved from the LPAIV strain either through a recombination event [6], [7] or a gradual increase in virulence over time through the insertion or substitution of basic amino acids at the HA cleavage site [8], [9].

Among water birds, mallards are of great interest due to their widespread distribution, reservoir for subtypes H1–H12, and ability to shed large amounts of virus with minimal pathology and disease [10], [11]. Mallards can also travel large distances and have been implicated as carriers of AIVs from one region to another [12], [13]. In mallards, it has been shown that the minimum duration of shedding decreases over a season of sampling among individual birds, and is likely due to transient immunity [14], but with limited data on actual strains causing infection, the concern remains that while infection and shedding continue to occur, the ability of LPAIV to mutate or evolve into HPAIV remains. Furthermore, some HP H5N1 viruses are non-pathogenic in mallards [11] or can become non-pathogenic through evolutionary adaptation in the duck host while remaining highly pathogenic to other domestic poultry. This would allow for the possibility of ducks transmitting the virus to other poultry without themselves suffering disease [15].

The relative roles of direct contact versus environmental contamination in the transmission of AIVs remains poorly understood; both mechanisms likely occur based on experimental and field studies [16], [17], [18], [19]. Understanding routes of transmission is important to modeling spread of virus [20], [21], [22]. AIVs have been shown to persist in water sources [23], [24] and may provide a source of contamination to other species sharing the same source. It has also been shown that over a 4 year period in Hong Kong, virus was isolated throughout the year from domestic ducks [25].

Common concerns with transmission of AIV from waterfowl to other species are evident when you observe interactions of multiple species, both domestic and wild, present within a single small farm, virtually anywhere in the world. Transmission to species such as rodents would not likely result in disease spread, but could be exploited to monitor disease incursion via serosurveillance; rats and mice are found in abundance on small farms and are of concern in their ability to move freely from outside into enclosures, and their propensity to eat and drink from common containers of poultry feed. Although rats are not considered reservoir hosts for influenza viruses, both laboratory and cotton rats have been shown to replicate unadapted avian and human influenza A viruses [26], [27], [28]. Similarly, pigeons are not generally considered an important host for transmission of influenza viruses, but are ubiquitous on small farms and undoubtedly exposed to these viruses on a routine basis. Their susceptibility to experimental infection with both LPAIV or HPAIV has been variable [29], [30], [31], [32].

It is clear that wild and domestic ducks harbor and shed influenza A viruses and recurrently interact in nature with a broad range of other avian and mammalian species to which they might transmit such viruses. Estimating the efficiency and importance of such multispecies transmission using epidemiological approaches is difficult. We therefore addressed this question by studying transmission of LP H5 and H7 viruses from infected ducks to other common animals in a quasi-natural laboratory environment designed to mimic a common barnyard.

## Materials and Methods

### Ethics Statement

All experiments were approved by the Institutional Animal Care and Use Committee of Colorado State University, Fort Collins, Colorado, USA, under approval number 09-168A.

### Animals

Mallard ducks and chickens were purchased from local producers at 2–4 months and 3–4 weeks of age respectively. Red-winged blackbirds and pigeons were captured locally. Sprague Dawley rats, 6–8 weeks of age were obtained from Charles River Laboratories. All animals tested negative for group-specific antibodies to influenza A virus by ELISA and strain specific antibodies (H5 and H7; <10) by hemagglutination inhibition (HAI) assay prior to infection.

### Viruses, Virus Assays and Serologic Assays

The viruses used in this study were A/Mallard/MN/346250/00 (H5N2) and A/Ruddy turnstone/ReedsBeachNJ/00 (H7N3). Both viruses were propagated to passage three in 10 day old specific pathogen free embryonated chicken eggs (Sunrise Farms, NY). Eggs were incubated at 37 C and allantoic fluid was harvested 48 hours after inoculation, aliquoted, and stored at −80 C until use. Both viruses were titrated by plaque assay. Briefly, plaque assays were performed on monolayers of MDCK cells (ATCC, Manassas, VA) in 6-well plates. The monolayers were washed with PBS, inoculated with sample, incubated 60 minutes at 37 C, then overlaid with 0.5% agarose in minimal essential medium containing 0.5% bovine albumin, antibiotics and TPCK trypsin (1 µg/ml). Following a 48 hour incubation at 37 C, a second overlay containing neutral red (33 mg/L) was applied and plaques were visualized and counted 4–6 hours later.

The challenge virus for each experiment was used to determine subtype-specific antibodies using the hemagglutination-inhibition assay [33], using sera treated with receptor destroying enzyme (Denka Seiken, Tokyo, Japan) as previously described [34]. Serial 2-fold dilutions of sera in PBS were prepared in 96-well V-bottom plates and mixed with 0.5% chicken red blood cells; titers of 10 or greater were considered positive. Group specific antibodies were detected using a commercial cELISA test (Flu DETECT® BE, Synbiotics Corporation, Kansas City, MO) based on detection of antibodies to a recombinant AIV nucleoprotein antigen. Experiments were carried out following the manufacturer's instructions. The manufacturer has validated this assay utilizing both chicken and duck sera.

### Barnyard Transmission Experiment

**Figure 1:**
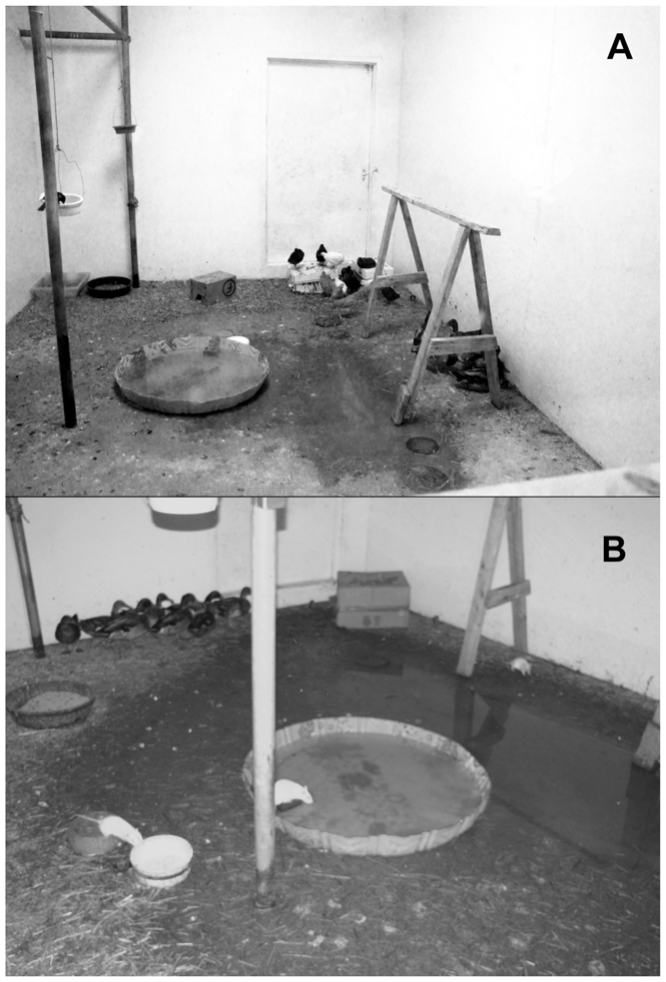
Barnyard room layout observed during the day (A) and at night (B).

Two independent experiments were conducted using different influenza viruses. In the first experiment, the barnyard contained 8 ducks, 8 chickens, 8 rats and 10 blackbirds. The second experiment consisted of 8 ducks, 8 chickens, 6 pigeons, 5 blackbirds and 7 rats. In both experiments, animals were allowed to freely range inside a room within an ABSL3 facility. The room had dimensions of 12 (width)×18 (length)×12 (height) feet and basic illumination was provided through a skylight in the roof. The barnyard rooms contained a plastic children's swimming pool (4 feet diameter, cut to 6 inches height) and two large bowls that contained commercial duck and chicken layer feed (Figure 1A). Three smaller bowls filled with songbird mixed grains were suspended approximately 6 feet off the floor from pipes that ran longitudinally across the room; these pipes also served as perches for the blackbirds and pigeons. Straw was spread across the floor sparsely and a sawhorse was present to provide additional perching opportunities for blackbirds and pigeons. Two or three cardboard boxes were provided as nest boxes for the rats. The pool was filled daily (but not emptied within the first week of the trial) with 5 gallons of water that had been sitting at room temperature and aerated with an aquarium pump for 24 hours to dechlorinate and thus prevent inactivation of any influenza virus [35]. This was also done to better mimic the natural state of water such as lakes and streams and farm water, which are typically not chlorinated. In the first experiment, night vision (infrared) cameras were installed in the room to allow monitoring of behaviors in the dark (Figure 1B).

On day 0 of each experiment, four ducks were removed from the barnyard room, placed in a separate room, and inoculated orally, intranasally and ocularly with 10^6^ PFU/0.5 ml of the respective virus. After four hours, the four inoculated ducks were returned to the barnyard where all animals were free to move about the room and interact. All eight ducks (4 inoculated and 4 non-inoculated) were sampled on days 0–7 by collecting oral and cloacal swabs into 2 ml BA-1 medium (MEM, 1% bovine serum albumin, 350 mg/L sodium bicarbonate, 50 ml/L 1 M Tris, pH7.6, 5 mg/L phenol red) supplemented with antibiotics (gentamicin 50 ug/ml, polymyxin B 100 U/ml, nyastatin 50 U/ml, penicillin, 100 U/ml and streptomycin 50 ug/ml), refrigerated and tested within 2 days for virus titer by plaque assay on MDCK cells; titers are reported as PFU/ml. The only barnyard animals to have daily swab samples collected for virus titration were ducks as we wished to minimize the stress on remaining barnyard species and use seroconversion as the determination of virus infection. All animals in the barnyard room were bled on days 0, 14, 21 and 28, and those sera were tested for seroconversion using HAI and ELISA.

Two to three water samples were collected daily before the addition of new water. Samples were collected by skimming the top of the pool with a tube, sediment by running the tube along the bottom of the pool to collect sample, and, if present, taking a sample of splashed water from the floor near the pool. To assist in interpreting pool water virus titers, we conducted an *in vitro* experiment in which pool water from a room containing non-infected ducks was spiked with the H5 or H7 viruses, maintained at room temperature and tested by plaque assay, as described above, at intervals up to 42 days.

### Direct Inoculation of Control Animals

For both experiments, groups of each of the animals in the barnyard except ducks were housed in cages in a separate room and directly inoculated with virus to determine the effects of known exposure. Chickens, pigeons, blackbirds and rats were inoculated intranasally with 10^6^ PFU in 0.1 ml. Once daily on days 0–7, oropharyngeal and cloacal swabs were collected from the birds, and oral swabs from rats; these samples were processed as described above for duck samples. Sera were collected on days 0, 14, 21, and 28, and tested for anti-influenza antibodies by ELISA and for challenge virus-specific antibodies by HAI.

### *In vitro* detection of virus

Water was collected from a pool that had non infected ducks swimming in it for 24 hours prior to water collection. This was done to mimic the natural state of the water from the barnyard study where water would also contain feces and food particles. Pool water was then placed in a 50 ml conical tube and spiked with either 1×10^6^ PFU/ml of H5N2 or H7N3 virus and placed at room temperature. A tube of the same water not spiked with virus served as the negative control and was collected and tested for virus. Samples were collected once daily on days 0 through 7 then weekly for 6 weeks. One ml aliquots were collected at each time point and stored at −80 until all samples were collected. Samples were then tested for virus titer utilizing the plaque assay.

## Results

Clinical signs of disease were not observed in any of the birds or rats in the barnyard environments nor among those caged and directly inoculated with either virus. Animals in the barnyard were observed several times daily. The ducks and chickens tended to cluster and move about in their own groups. Blackbirds and pigeons spent much of their time perched above the floor, but were frequently observed walking on the floor or perched on the side of the pool. All of the birds and rats were observed drinking from the pool and eating out of common feed bowls on the floor. The rats were almost never seen out of their houses during daylight, but were confirmed by video to be exceptionally active in running around the room and through the pool of water during the dark (Figure 1B).

### Infection and Transmission: H5N2 virus

**Figure 2:**
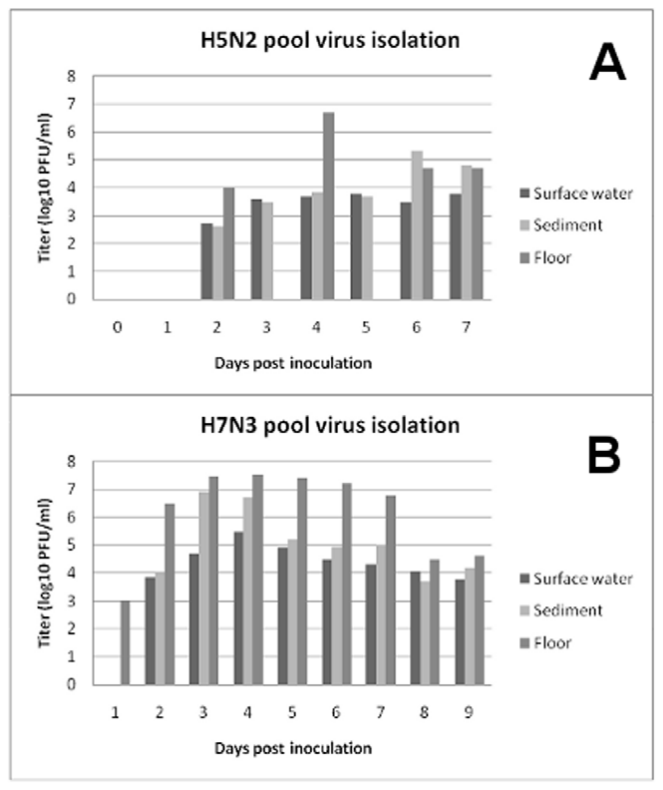
Accumulation of H5N2 (A) and H7N3 (B) viruses in barnyard pool water. Water samples skimmed from the surface of the pool, off the bottom (sediment-rich) or splashed onto the floor were assayed for infectious virus by plaque assay on MDCK cells.

**Figure 3:**
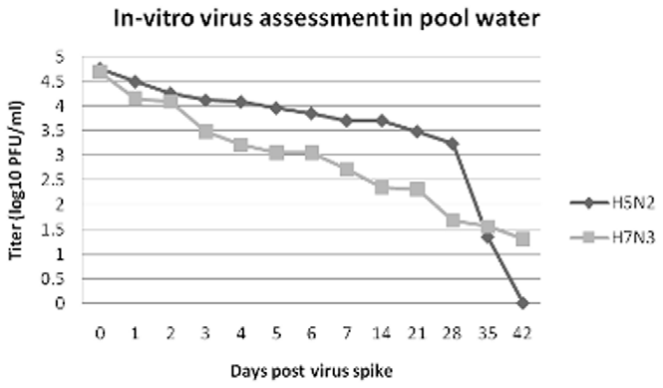
Survival of H5N2 and H7N3 viruses added to duck pool water and maintained at ambient temperature. Water from a pool used by non-infected ducks was spiked with virus, sampled over time and assayed by plaque assay on MDCK cells.

Virus was shed by all four inoculated ducks and transmitted to all four contact ducks either through direct contact or environmental contamination of the floor and shared pool (Table 1). As would be expected with LPAIV in ducks, virus was shed to higher titers by the cloacal versus oral routes. Contact ducks did not begin shedding detectable virus until at least 1 day after inoculated ducks began shedding. Detectable shedding of virus from ducks ended on day 5 post inoculation. H5N2 virus was first detectable in sampled water on day 2 post inoculation and continued until day 7 which was the last day samples were collected before the pool was emptied completely and refilled (Figure 2A). Titers of virus were comparable in all three samples on each day except the floor sample from day four was 100-fold greater than either the sediment or surface pool water sample, likely due to a concentration of feces in that area on that day. *In vitro* testing of H5N2 virus stability in pool water demonstrated a steady decline in virus titer with viable virus detected out to day 35 (Figure 3).

**Table 1:** Virus shedding from inoculated and contact ducks.

				Virus titer in swab sample (log_10_ PFU/ml)						
Virus	Exposure	Sample*	Duck	1§	2	3	4	5	6	7
H5N2	Inoculated	CLO	2	<1.0	<1.0	<1.0	1.0	2.0	<1.0	<1.0
			3	<1.0	4.7	2.8	1.5	<1.0	<1.0	<1.0
			4	<1.0	3.0	3.6	3.1	2.9	<1.0	<1.0
			6	<1.0	5.9	3.8	3.0	2.0	<1.0	<1.0
		OP	2	<1.0	1.3	<1.0	<1.0	<1.0	<1.0	<1.0
			3	2.3	1.3	<1.0	1.0	<1.0	<1.0	<1.0
			4	<1.0	<1.0	<1.0	2.0	<1.0	<1.0	<1.0
			6	<1.0	1.0	<1.0	1.0	<1.0	<1.0	<1.0
	Contact	CLO	1	<1.0	<1.0	3.1	4.6	3.1	<1.0	<1.0
			5	<1.0	<1.0	4.6	3.3	2.7	<1.0	<1.0
			7	<1.0	<1.0	4.1	3.9	3.3	<1.0	<1.0
			8	<1.0	<1.0	3.0	4.3	2.9	<1.0	<1.0
		OP	1	<1.0	<1.0	<1.0	1.0	<1.0	<1.0	<1.0
			5	<1.0	<1.0	1.9	1.5	1.5	<1.0	<1.0
			7	<1.0	1.5	1.8	2.3	<1.0	<1.0	<1.0
			8	<1.0	<1.0	1.5	<1.0	1.8	<1.0	<1.0
H7N2	Inoculated	CLO	1	<1.0	4.4	3.7	2.5	<1.0	<1.0	<1.0
			2	3.3	3.5	3.0	2.9	1.0	1.6	<1.0
			3	2.3	3.5	3.0	2.5	1.3	<1.0	<1.0
			7	<1.0	3.5	2.9	3.3	2.3	<1.0	<1.0
		OP	1	1.3	3.0	1.5	2.4	1.7	2.3	<1.0
			2	2.3	2.5	2.5	2.9	1.3	1.0	<1.0
			3	1.0	<1.0	1.0	3.3	1.0	<1.0	<1.0
			7	2.7	2.3	2.6	2.9	1.7	<1.0	<1.0
	Contact	CLO	4	<1.0	2.6	3.6	3.3	<1.0	<1.0	<1.0
			5	<1.0	3.3	3.0	3.6	2.0	<1.0	1.0
			6	<1.0	4.6	3.7	2.0	3.7	1.0	<1.0
			8	<1.0	6.3	6.5	3.5	3.7	1.0	<1.0
		OP	4	<1.0	2.5	1.8	1.6	2.5	<1.0	<1.0
			5	<1.0	1.0	2.0	1.6	1.5	<1.0	<1.0
			6	<1.0	2.3	2.0	<1.0	1.3	<1.0	<1.0
			8	<1.0	2.0	2.3	2.3	2.9	<1.0	<1.0

*Swab samples are cloacal (CLO) or oropharygeal (OP).

§ Numbers represent days post challenge.

In the direct inoculation of control animals experiment, 83% of chickens and 100% of blackbirds shed detectable virus orally on days 1 through 5 and 1 through 6 respectively (Table 2); small amounts of virus were detected sporadically on cloacal swabs from one chicken on day 4. Virus was not recovered from rats or pigeons that were directly inoculated with virus.

**Table 2:** Virus isolation from oropharygeal swabs taken from directly inoculated control animals.

Virus	Species	Number Shed	Days shed	Peak day of shedding	Peak virus titer (log10 PFU/ml)
H5N2	Chicken	5/6	1–5	3	2.6
	Blackbird	6/6	1–6	2	3.6
	Pigeon	0/6	NA	NA	NA
	Rat	0/6	NA	NA	NA
H7N3	Chicken	5/6	1–7	2	2.3
	Blackbird	4/4	1–7	4	4.0
	Pigeon	1/6	1–3	1	3.5
	Rat	0/6	NA	NA	NA

In order to determine infection rates of all animals exposed in the barnyard or directly inoculated, sera were collected on days 0, 14, 21 and 28 post-inoculation or exposure.

For the contact chickens in the barnyard we observed seroconversion rates in chickens of 63% by HAI and 100% by ELISA (Table 3). None of the barnyard contact rats and blackbirds seroconverted by either HAI or ELISA. For the directly inoculated control animals, there was 100% seroconversion in the chickens and rats by both HAI and ELISA but only 50% (1/2) in the blackbirds by HAI (Table 3). The ELISA failed to detect antibody in any blackbird, regardless of HAI titer or virus isolation. One caveat to the experiment with the blackbirds is that repeated daily handling to obtain the cloacal and oral swabs proved too stressful and 3 of the 6 blackbirds died due to non-influenza complications before serum collection was initiated on day 7.

**Table 3:** Seroconversion following virus exposure in directly-inoculated (caged) and contact (barnyard) animals.

Species and exposure	H5N2 virus		H7N3 virus	
	HAI*	ELISA	HAI	ELISA
Duck, Inoculated	3/4 (75%)§	4/4 (100%)	1/4 (25%)	4/4 (100%)
Duck, Contact	3/4 (75%)	4/4 (100%)	2/4 (50%)	4/4 (100%)
Chicken, Inoculated	6/6 (100%)	6/6 (100%)	4/6 (67%)	6/6 (100%)
Chicken, Contact	5/8 (63%)	8/8 (100%)	8/8 (100%)	8/8 (100%)
Blackbird, Inoculated	1/2 (50%)*	0/2 (0%)*	1/1 (100%)*	0/1 (0%)*
Blackbird, Contact	0/10 (0%)	0/10 (0%)	4/5 (80%)	0/5 (0%)
Pigeon, Inoculated	0/6 (0%)	5/6 (83%)	0/6 (0%)	2/6 (33%)
Pigeon, Contact	ND	ND	0/6 (0%)	5/6 (83%)
Rat, Inoculated	6/6 (100%)	6/6 (100%)	4/5 (80%)	5/5 (100%)
Rat, Contact	0/8 (0%)	0/8 (0%)	0/7 (0%)	6/7 (86%)

*HAI titer ≥10 were considered positive.

§ Number of birds positive/total (% positive) at any one timepoint from days 14, 21, or 28.

### Infection and Transmission: H7N3 Virus

Virus was shed by all four inoculated ducks and transmitted to all four contact ducks (Table 1). Virus was shed longer and to slightly higher titers than with the H5N2 virus isolate which was only detectable to day 5. Virus titers from oral samples were also higher than oral viral titers seen in the H5N2 experiment. As seen with the H5N2 experiment, contact ducks did not shed detectable virus until at least one day after inoculated ducks began shedding, as would be expected in cases of transmission (Table 1). Virus shedding was detected in at least one duck on all days 1 through 7. For the H7N3 experiment we extended the number of days of water sample collection from 7 days to 9 before cleaning out the pool. H7N3 virus was detected in samples from day 1 through day 9, with floor samples showing the highest levels of virus at all time points. (Figure 2B) H7N3 virus was also tested *in vitro* for stability, and behaved similarly to the H5N2 virus with viable virus detected out to day 42 (Figure 3).

Virus isolation was also performed on all control animals directly inoculated with H7N3 virus, including blackbirds, pigeons, chickens and rats. Virus was isolated from oropharygeal swabs at various time points from 83% of chickens, 16% of pigeons, and 100% of blackbirds (Table 2), but was not isolated from any of the oral swabs collected from rats.

Transmission rates in the barnyard were assessed serologically on days 0, 14, 21 and 28 post inoculation. Only 25% of virus-inoculated ducks seroconverted based on HAI results but 100% were positive by ELISA, correlating with the fact that 100% shed virus to varying degrees in the experiment (Table 3). Of the four contact ducks, 50% seroconverted by HAI and 100% by ELISA. Also in the barnyard, contact chickens, rats, blackbirds, and pigeons seroconverted at 100%, 0%, 80%, and 0% respectively as detected by HAI and 100%, 86%, 0%, and 83% by ELISA (Table 3).

For the directly inoculated chickens, rats, blackbirds, and pigeons we observed seroconversion rates of 67%, 80%, 100%, and 0% by HAI respectively, and 100%, 100%, 0%, and 33% by ELISA respectively (Table 3). As with the H5N2, the experimentally infected blackbirds did not all survive to day 7 for serum collection so the percentage represents only one of the original four blackbirds that survived to the end of day 21 (Table 3).

## Discussion

In both barnyard experiments, introduction of recently-infected mallards was followed rapidly by infection and shedding of virus by contact ducks, and accumulation of substantial quantities of virus in water from the shared pool. Based on detection of seroconversion, ducks infected with either virus efficiently transmitted the virus to other species either through direct contact, which would be most likely with the contact ducks, or through contamination of the environment such as the pool and floor water where high virus titers were recovered in titers high enough to infect the other species. The H7N3 virus was transmitted to a large fraction of other animals in the room, including blackbirds, pigeons and rats, but transmission of the H5N2 virus to blackbirds and rats was not detected. This apparent difference in cross-species transmission may reflect, in part, differences in transmissibility between the two viruses, but it is more likely that transmission of the H7 virus was enhanced due to its higher magnitude and duration of shedding, and higher levels of accumulation in the shared water source compared to that of the H5 virus. As anticipated, neither of the two viruses induced noticeable signs of disease in any of the exposed animals, including those directly inoculated with virus.

The high titers of virus that accumulated in water of the shared barnyard pools undoubtedly were in excess of what might typically be expected in natural situations involving wild mallards, but may not be altogether unrealistic for low pathogenic AIVs in small bodies of water. Moreover, it seems likely that such titers may occur in ponds associated with high density domestic duck production facilities, although studies attempting to measure virus titers in such situations are lacking with ducks. The presence of environmental AIV in water habitats of turkeys supports the need for increased environmental sampling along with avian surveillance studies [17]. It is evident however, from this and other experimental studies, that efficient transmission via contaminated water can occur among ducks and between ducks and other birds [19], [36].

Rats were included in the barnyard transmission experiments because they or other rodents are inevitably present on small farms, sometimes in large numbers, are in direct contact with ducks and poultry and are able to move freely among enclosures. Live markets in Asia, where H5N1 influenza is prevalent, are an additional setting where large numbers of rats live in close contact with ducks and chickens [28]. The role of rodents in facilitating spread of AIVs is essentially unknown. Rats housed in the H7N3 contaminated barnyard room seroconverted to that virus, as did caged rats inoculated with both H5N2 and H7N2 viruses. Shedding of neither virus from infected rats was detected, supporting the idea that they do not play a significant role in transmission to other species.

Sero-surveys in natural settings [37], [38], [39] as well as in experimental studies [40] have provided valuable insights into the infection rates of mallards and other wild birds. In the current study, seroconversion was used to evaluate virus transmission among the barnyard animals and to assess infection in the animals directly inoculated with virus; further, it would have been extremely stressful to capture the blackbirds and pigeons daily to obtain samples for virus isolation. Results obtained from the nucleoprotein blocking ELISA more accurately reflected the results of virus isolation and known virus exposure than did the HAI test, and allowed detection of virus transmission to rats and pigeons in the H7 barnyard trial. An interesting exception to this finding was that the ELISA consistently failed to detect antibodies to either influenza virus in blackbird sera despite their being positive by HAI testing. Results reported here suggest that multiple serologic tests are necessary to accurately conduct serosurveillance for influenza viruses when diverse species are involved. Additional research to identify factors responsible for these serologic discrepancies would clearly be beneficial to surveillance efforts and allow an enhanced understanding of the ecology and evolution of AIVs worldwide.

Both LPAIVs tested efficiently spread from ducks to chickens within the shared environment, and a majority of chickens directly inoculated with these viruses shed them at reasonable levels from the intestinal tract; we did not test whether chicken-to-chicken transmission occurred. Transmission of LPAIV from ducks to chickens, if accompanied by mutations in the hemagglutinin gene encoding the HA-1∶HA-2 cleavage site, could lead to generation of a HPAIV and subsequent outbreak in poultry. It is not known whether either of these viruses replicating in chickens, blackbirds, or pigeons might evolve and adapt to those hosts, allowing the new host to better transmit the virus or whether the virus would encounter a dead end in the new host.

Small farms, live and wet markets, and many poultry shows provide abundant opportunities for interactions among free ranging and domestic species which may result in transmission and perpetuation of AIVs, particularly when ducks are involved. The studies reported here indicate that introduction of ducks infected with LPAIV into a room designed to mimic a typical barnyard resulted in efficient dissemination of virus to a number of other species, including other birds and rodents. This model system should be extended to investigate more refined questions, such as transmission from passerines to ducks or chickens, multiround transmission, and transmission involving additional viruses, including H5N1 AIV.
